# Nonsurgical Pneumoperitoneum: A Rare Thoracoabdominal Complication of Asthma Exacerbation

**DOI:** 10.1002/rcr2.70294

**Published:** 2025-07-24

**Authors:** Takuya Nagahara, Kazuhiko Iwasaki, Saya Tsukida, Akihito Okazaki

**Affiliations:** ^1^ Department of Respiratory Medicine Kaga Medical Center Kaga Ishikawa Japan; ^2^ Department of Respiratory Medicine Kanazawa University Graduate School of Medical Sciences Kanazawa Ishikawa Japan

**Keywords:** asthma exacerbation, free air, nonsurgical pneumoperitoneum

## Abstract

Asthma‐related nonsurgical pneumoperitoneum is extremely rare. This case highlights that even without abdominal symptoms, free air may result solely from asthma exacerbation, and conservative treatment without fasting can be sufficient after surgical causes are excluded.

## Graphical abstract

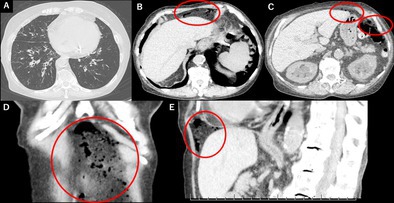

An 82‐year‐old woman with bronchial asthma presented with dyspnea and cough and was diagnosed with respiratory failure. Chest computed tomography (CT) revealed diffuse bronchial‐wall thickening consistent with asthma exacerbation (Figure 1A) and free air under the diaphragm, without any intra‐abdominal pathology (Figure 1B–E). She remained haemodynamically stable and had no abdominal symptoms such as tenderness.

**FIGURE 1:**
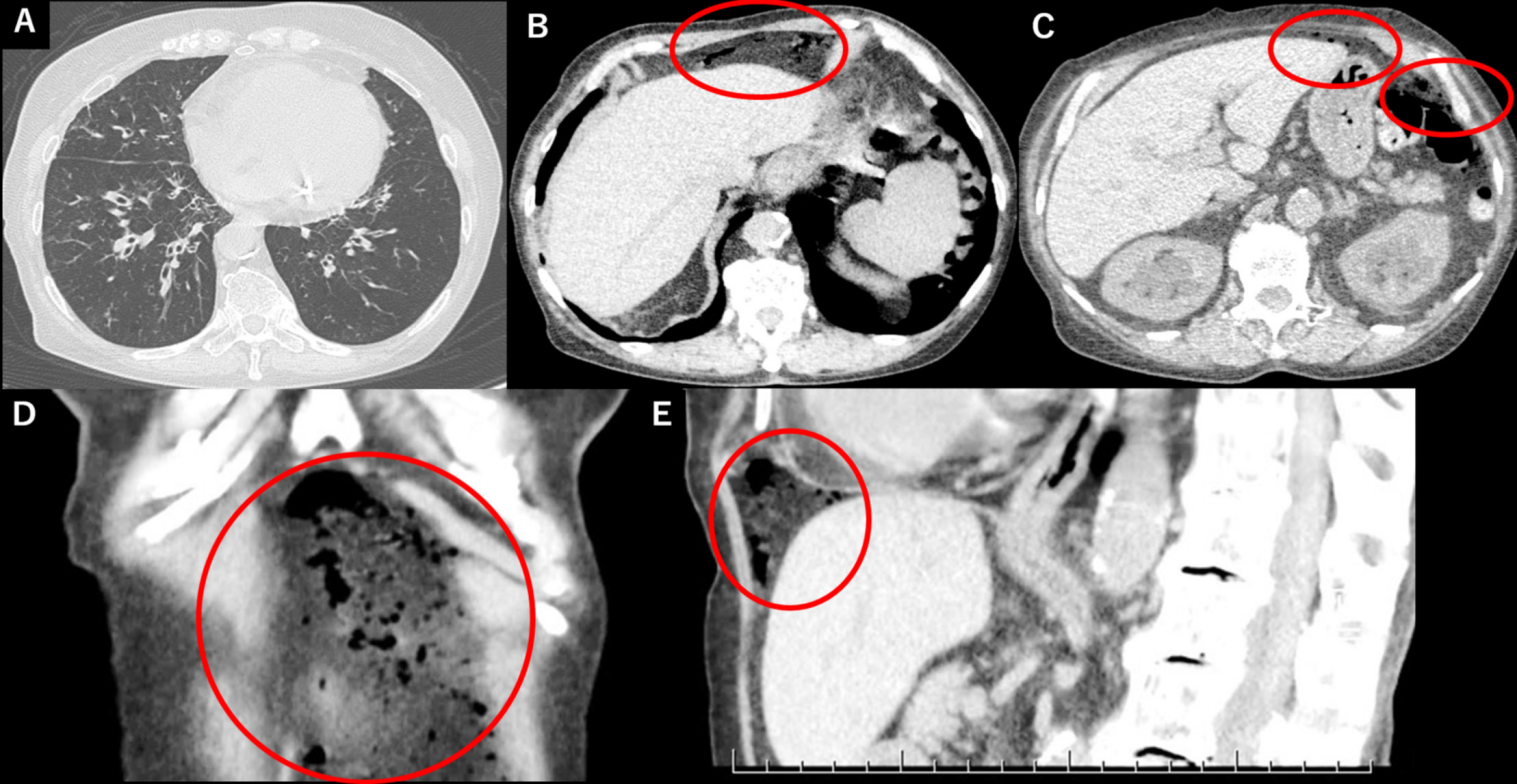
Initial computed tomography (CT) images on admission. (A) Axial chest CT in lung window showing diffuse bronchial‐wall thickening, consistent with asthma exacerbation. (B, C) Axial, (D) coronal, and (E) sagittal abdominal‐window CT images show localised intraperitoneal free air near the diaphragm (circles). No evidence of pneumothorax, pneumomediastinum, or intra‐abdominal pathology is observed.

Based on the clinical course, nonsurgical pneumoperitoneum (NSP) associated with asthma exacerbation was diagnosed. She was treated conservatively with rest, systemic corticosteroids and inhaled bronchodilators. Follow‐up CT on the next day showed complete resolution of the pneumoperitoneum (Figure 2A–D).

**FIGURE 2:**
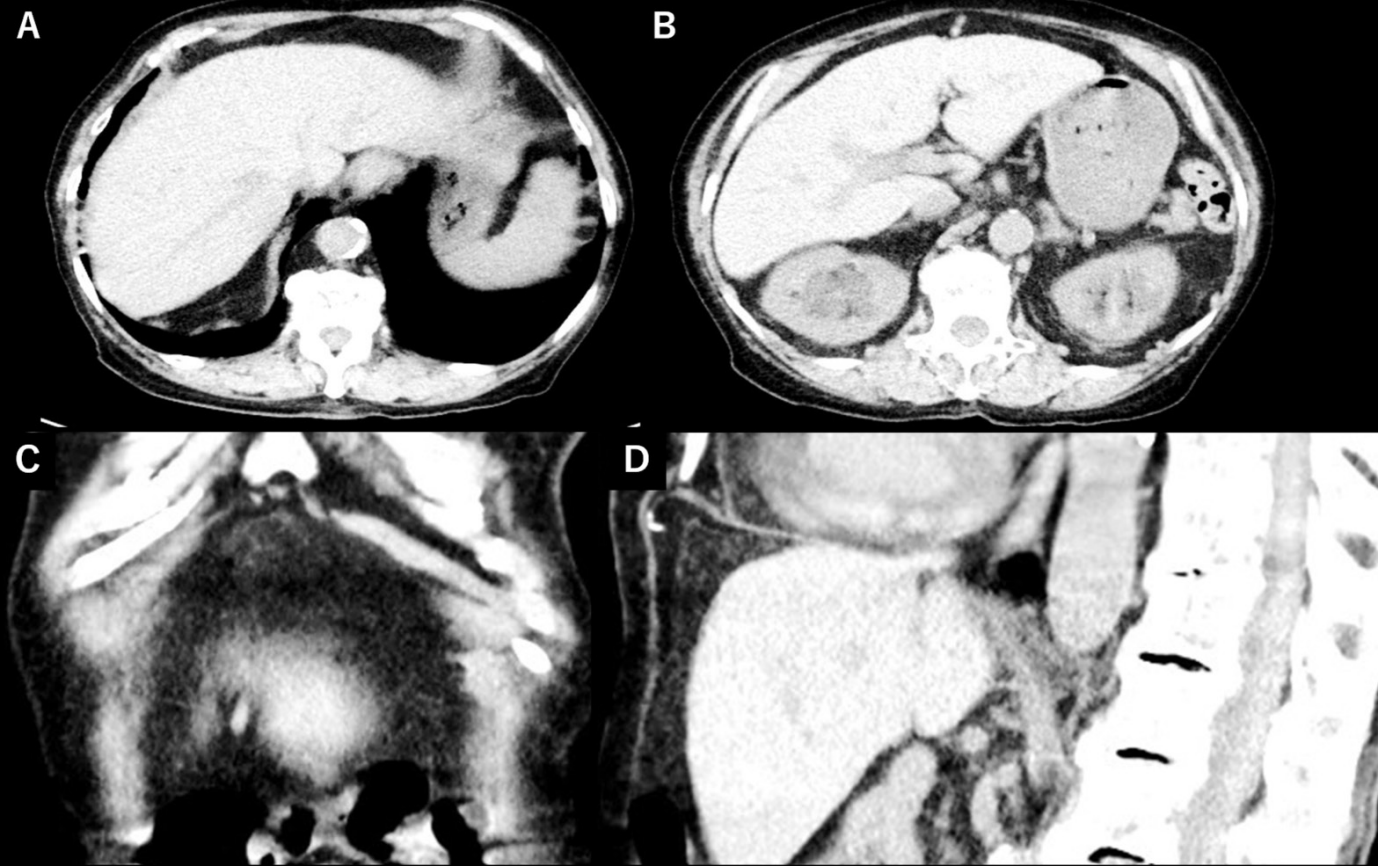
Follow‐up CT images obtained the day after admission, corresponding to the same anatomical planes as in Figure 1B–E. (A, B) Axial, (C) coronal, and (D) sagittal abdominal‐window CT images show complete resolution of the previously noted intraperitoneal free air. These findings parallel the patient's clinical improvement following conservative treatment.

NSP accounts for approximately 10% of all pneumoperitoneum cases and is typically associated with abdominal interventions. Thoracic causes, including mechanical ventilation or cardiopulmonary resuscitation, are rare [1]. Although one case of asthma‐associated NSP has been reported [2], that case involved abdominal symptoms and did not clearly arise in the context of an asthma exacerbation. In contrast, our case developed solely in the setting of an asthma attack, without abdominal complaints or procedures, and was successfully managed without fasting or surgical consultation. This highlights the possibility of conservative management in carefully selected patients. Clinicians should consider this rare presentation and exclude surgical emergencies.

## Author Contributions

T.N. and K.I. wrote the initial draft of the manuscript and were responsible for the drafting and image modification. T.N., K.I., S.T. and A.O. were directly involved in treatment, critically revised the manuscript, and approved the final version.

## Consent

Written informed consent was obtained from the patient for the publication of this report in accordance with the journal's patient consent policy.

## Conflicts of Interest

The authors declare no conflicts of interest.

## Data Availability

Research data are not shared.
